# Using procalcitonin to guide antimicrobial duration in sepsis: asking the same questions will not bring different answers

**DOI:** 10.1186/cc13870

**Published:** 2014-05-13

**Authors:** Jorge IF Salluh, Vandack Nobre, Pedro Povoa

**Affiliations:** 1 D’Or Institute for Research and Education, Rio de Janeiro 22281-100, Brazil; 2 Postgraduate Program, Instituto Nacional de Câncer, Rio de Janeiro 20231-092, Brazil; 3 Graduate Program in Infectious Diseases and Tropical Medicine, Department of Internal Medicine, School of Medicine and Hospital das Clínicas, Universidade Federal de Minas Gerais, Belo Horizonte 30130-090, Brazil; 4 Polyvalent Intensive Care Unit, Hospital de São Francisco Xavier, Centro Hospitalar de Lisboa Ocidental, CEDOC, Faculty of Medical Sciences, New University of Lisbon, Lisboa 1449-005, Portugal

## Abstract

Severe sepsis is a major healthcare problem and the early initiation of antimicrobials is one of the few measures associated with improved outcomes. However, antibiotic overuse is an increasing problem in critical care. Of several potential biomarkers for antibiotic stewardship, procalcitonin represents the most widely studied and validated. In this commentary we address the current literature on the use of biomarkers to guide antimicrobial therapy in the critically ill and discuss its limitations and future directions.

## 

In a previous issue of *Critical Care*, Prkno and colleagues report a meta-analysis of the current literature evaluating the role of procalcitonin (PCT) to guide the duration of antimicrobial therapy in patients with severe sepsis and septic shock admitted to the ICU [1]. In these patients early initiation of antimicrobials is one of the few measures associated with improved outcomes in this high mortality scenario [2]. However, overuse of antimicrobials - related either to missed opportunities to reduce the spectrum of activity or to unnecessarily long treatment regimens - has become an increasing problem in the past decades. Besides being associated with worse outcomes, antimicrobial resistance is becoming even more worrisome.

Clinical judgment remains the most valuable tool when decisions regarding the use of antibiotics are taken, but with important shortcomings. While features of disease severity at presentation may be quite nonspecific, the characteristics of clinical response to therapy are usually inaccurate and occur late in the course of disease. In such a scenario, biomarkers have been proposed as important tools to aid the decision-making process. Of several potential candidates, PCT represents the most widely studied and validated.

## What becomes clearer from current studies in sepsis

Several systematic reviews have addressed the issue of PCT-guided therapy. Prkno and colleagues [1] focused on critically ill patients with severe sepsis or septic shock. By doing so, they cleverly avoided biases related to the heterogeneity of studied patients, specifically the wide variation of severity that may exist between infected individuals (for example, from mild to severe community-acquired pneumonia) [3]. Using these inclusion criteria, seven randomized clinical trials were enrolled, totaling 1,075 patients. As their main conclusions, Prkno and colleagues provide a clear message for ICU physicians: we can safely reduce the duration of antimicrobial therapy in patients with severe sepsis or septic shock using PCT-guided protocols (hazard ratio 1.27, 95% confidence interval 1.01 to 1.53). Nevertheless, the authors acknowledge the many limitations of their study (for example, heterogeneity of PCT protocols) and emphasize that further information on major outcomes, such as length of stay and mortality, is required from future studies.

## Limitations of current studies and their clinical implications

Despite their important contribution, most trials evaluating PCT-guided antibiotic therapy present several limitations that preclude their safe extrapolation to the clinical decision-making process [3]. Namely, the high rate of patient exclusion (reaching >80% in the Svoboda and colleagues trial [4]) and the high rate of algorithm overruling (reaching >50% in the PRORATA trial [5]) disregard the impact of renal failure as well as renal replacement therapy on PCT levels, and above all the heterogeneity of duration of antibiotic therapy in the controls. Contrary to Prkno and colleagues’ opinion, since 2003 [6,7] it has been clear that the duration of antibiotic therapy could be safely reduced in critically ill patients to 6 to 8 days. Consequently, this duration of therapy should be used as the best care in clinical trials. Besides, some infections (for example, infectious endocarditis, and nosocomial infections due to *Pseudomonas aeruginosa* or *Acinetobacter baumannii*) were not systematically evaluated. Finally, the costs associated with daily measurement of PCT in all ICU patients should not be ignored, since we have cheaper and widely available alternatives with proven safety and efficacy [8].

## Improving the design of future studies and the current clinical use of biomarkers in sepsis

With the previously mentioned shortcomings in mind, it is legitimate to ask how can we properly use biomarkers such as PCT and C-reactive protein (CRP) to guide antimicrobial therapy (initiation and duration) in severe sepsis. First, we believe that future clinical trials should use less strict entry criteria that would better reflect our real-life ICU patients with sepsis. Second, a great deal of effort must be made to conduct multicenter studies involving large numbers of patients, ideally in different regions of the world. Lastly, biomarker-guided strategies must be tested against a comparator that actually reflects the 'best care' (that is, implementation of the best available evidence), and not the highly variable 'standard care'. In our opinion, it means comparing PCT algorithms with control treatment in which the maximum durations of antibiotic therapy is setup in 7 days, or even in 5 days (for example, severe sepsis without shock) [6,7,9]. Until these studies are performed and their results become available, clinicians should perhaps use a 'double-trigger' strategy as proposed by Oliveira and colleagues [8]. In all patients, antibiotics were stopped according to the clinical course and either decreases in biomarker levels, according to an algorithm, or the completion of 7 days of treatment, whichever came first. In this single-center study evaluating patients with severe sepsis and septic shock, PCT or CRP were similarly effective to ensure early interruption of antibiotics (day 4/5).

**Figure 1:**
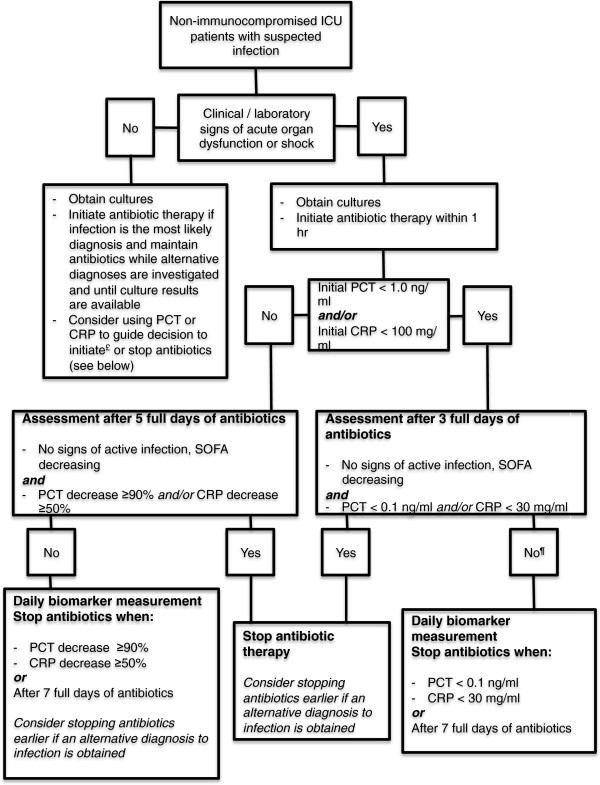
**Use of C-reactive protein and procalcitonin to guide antimicrobial therapy in critically ill patients.** C-reactive protein (CRP) was tested only in a single center trial with predominantly medical ICU patients. This flowchart does not apply to immune-compromised patients (for example, febrile neutropenia) or to patients with infections requiring long-term antibiotic therapy (for example, infectious endocarditis, cerebral abscess, bacteremia due to *Staphylococcus aureus*). ^£^Most trials investigating procalcitonin (PCT)-guided protocols tested the role of this marker in guiding the decision of antibiotic interruption. Initiating antibiotics for all critically ill patients with suspected infection is probably the safest decision, regardless the levels of laboratory biomarkers. However, this decision must be reassessed daily. PCT and CRP are proposed as additional tools to diagnose infection, and different cutoff levels have been proposed in the literature. ^¶^Consider stopping antibiotics before day 7 in patients with no proven infection (for example, negative cultures) regardless the levels of CRP or PCT. SOFA, Sequential Organ Failure Assessment.

To achieve safe and efficient short-course antimicrobial therapy in severe sepsis we propose an algorithm that may aid clinicians in their decision-making process (Figure 1). Using such a protocol, which remains to be validated in multicenter studies, we would be applying two very sound and validated concepts: patients with a fast response pattern to antibiotic therapy (early and substantial decrease in biomarker levels) have better outcomes [10-12]; and most cases of severe infections may be treated with 7 days of antibiotic therapy [6,7,9].

## Abbreviations

CRP: C-reactive protein; PCT: Procalcitonin.

## Competing interests

PP has an unrestricted research grant from ThermoFisher Scientific and Virogates. The other authors state that they have no competing interests.
